# Low‐value care in musculoskeletal health care: Is there a way forward?

**DOI:** 10.1111/papr.13142

**Published:** 2022-09-15

**Authors:** Jan Hartvigsen, Steven J. Kamper, Simon D. French

**Affiliations:** ^1^ Department of Sports Science and Clinical Biomechanics, Center for Muscle and Joint Health University of Southern Denmark Odense M Denmark; ^2^ Chiropractic Knowledge Hub Odense M Denmark; ^3^ School of Health Sciences University of Sydney Sydney New South Wales Australia; ^4^ Nepean Blue Mountains Local Health District Penrith New South Wales Australia; ^5^ Department of Chiropractic Macquarie University Sydney New South Wales Australia

**Keywords:** back pain, clinical guidelines, evidence‐based practice, low‐value care, osteoarthritis

## Abstract

**Background:**

Low‐value care that wastes resources and harms patients is prevalent in health systems everywhere.

**Methods:**

As part of an invited keynote presentation at the Pain in Motion IV conference held in Maastricht, Holland, in May 2022, we reviewed evidence for low‐value care in musculoskeletal conditions and discussed possible solutions.

**Results:**

Drivers of low‐value care are diverse and affect patients, clinicians, and health systems everywhere. We show that low‐value care for back pian, neck pain, and osteoarthritis is prevalent in all professional groups involved in caring for people who seek care for these conditions. Implementation efforts that aim to reverse low‐value care seem to work better if designed using established conceptual and theoretical frameworks.

**Conclusion:**

Low‐value care is prevalent in the care of people with musculoskeletal conditions. Reducing low‐value care requires behaviour change among patients and clinicians as well as in health systems. There is evidence that behaviour change can be facilitated through good conceptual and theoretical frameworks but not convincing evidence that it changes patient outcomes.

## LOW‐VALUE CARE IS PREVALENT IN HEALTH SYSTEMS

Low‐value care is defined as health services that confer little or no benefit to patients or where risk of harm exceeds probable benefit, according to best available evidence.^1^ Low‐value care is common across health systems globally and includes ineffective screening programs, unnecessary diagnostic testing and imaging, ineffective and harmful treatments, and inefficient organization of health systems.^2^, ^3^ It is estimated that only around 60% of services are in line with best available evidence, 30% is waste, duplication, or low value, and 10% is harmful.^4^ Low‐value care is not a trivial issue; it adds cost and consumes resources, causes iatrogenic harm, and impedes delivery of high‐value care that reliably provides health benefits for individuals and populations.^5^

Drivers of low‐value care are numerous. In some circumstances clinicians act according to their own financial benefit rather than the patient's best interests.^6^ For example, provision of ineffective services can be motivated by commercial ties to ancillary service facilities or to pharmaceutical and medical device industries.^7^, ^8^ Clinician knowledge, assumptions, and bias also play a role in provision of low‐value care. Clinicians may be unfamiliar with best practice clinical guidelines or choose to disregard recommendations when evidence contradicts their training, professional identity, or perceived clinical experience.^9^, ^10^ The quality of the patient–clinician relationship is important. For example, when a patient distrusts the clinician and demands further testing or treatment, or when a clinician orders testing or imaging to protect against litigation as part of defensive medicine.^11^ Further, the way health systems are organized can promote low‐value care. Hospital income may be tied to unnecessary procedures, profits of private insurance companies sometimes increase with consumption of health care, and pharmaceutical companies may subsidize patient payments in order to increase patient demand.^12^ Final, perverse incentives exist in healthcare systems when interventions discouraged in evidence‐based guidelines (eg, opioids, imaging, and surgeries) are widely available and publicly funded, whereas recommended treatments (eg, physical and psychological therapies) are not.^13^

In this paper, we describe what high‐value care should look like according to guideline recommendations for people with musculoskeletal conditions (MSK) and provide examples of low‐value care. We argue the need for behavior change among patients, clinicians, and in healthcare systems, and we present models for implementation that might facilitate delivery of high‐value care. This paper is not a systematic review of the evidence; however, in preparation, we performed repeated literature searches of PubMed and Scopus to identify guideline recommendations for treatment of common MSK conditions (back pain, neck pain, and osteoarthritis), as well as systematic reviews or primary studies of evidence regarding adherence to these recommendations.

## GUIDELINE RECOMMENDED CARE

Clinical practice guidelines across MSK conditions provide several consistent recommendations, including the following.^14^, ^15^

- patient‐centered care containing information and education about the condition;
- consideration of psychosocial factors as appropriate for improving pain and function;
- encouraging people to remain at work;
- physical activity and exercise interventions;
- manual therapy only as an adjunct to other treatments;
- pharmacological options for short‐term pain relief with careful consideration of potential harm harms;
- advice to not perform routine imaging; and
- high‐quality non‐surgical care prior to surgery

Specifically, for low back pain, there is broad consensus that patients should initially receive non‐pharmacological care.^16^, ^17^, ^18^ Recommendations in guidelines for neck pain are similar to those in low back pain guidelines, although oral and topical analgesics are recommended as first‐line treatments.^16^ For people with hip or knee osteoarthritis, provision of exercise programs and support for weight management is emphasized.^19^, ^20^

## NON‐GUIDELINE RECOMMENDED CARE IS PREVALENT

There is overwhelming evidence that many patients receive care that does not reflect guideline recommendations, a pattern that is not confined to particular health professions. Kamper et al systematically reviewed studies describing usual care for people with back pain and found that if patients sought care from general practitioners, fewer than 20% of patients received evidence‐based information and advice, around 25% received referral for imaging, and between 20% and 30% received a prescription for opioids. When care is delivered in emergency departments, 30% received imaging and up to 60% were prescribed opioids. Furthermore, there is inconsistent provision of education and advice regarding maintenance of physical activity.^21^ In a systematic review including 94 primary studies evaluating whether physical therapists provided care consistent with guidelines for people with back pain, Zadro et al found that approximately 40% did not.^22^ Amorin‐Woods demonstrated that chiropractors provided inappropriate care to back pain patients 30% of the time.^23^ In Australia, an audit of online information on clinic webpages and on Facebook revealed that 72% of chiropractors and 61% of physiotherapists had breaches of advertising guidelines when marketing their practice and services to patients and the public.^24^ Further, a scoping review of the chiropractic literature demonstrated that adherence to imaging guidelines is sub‐optimal.^25^

Hagen et al reviewed studies that dealt with primary care for knee or hip osteoarthritis and found that less than half received an assessment of their pain and/or function, and fewer than 40% received recommendations for exercise or were offered education and self‐management support.^26^ Thorlund et al scrutinized prescription registries in Sweden and found that among more than 8000 people with newly diagnosed osteoarthritis of the knee or hip, more than half of incident opioid prescriptions were prescribed inappropriately according to national treatment guidelines.^27^ Nationwide reviews of health systems indicators show that just 45% of Australians,^28^ and less than 30% of UK^29^ patients with OA receive evidence‐based care.

Surgery is arguably the most radical treatment for any MSK disorder. Apart from injuries, the most common reason for orthopedic surgery is chronic pain related to osteoarthritis, back pain, or neck pain.^30^ Justification for these surgeries often relies on structural or mechanistic rationales, which ignores the influence of psychological and social factors on chronic pain and the poor link between degenerative changes and pain. For example, structural pathology was not related to patient‐reported pain and function in Danish patients undergoing meniscal surgery in the knee,^31^ and Brinjiki et al systematically reviewed 33 original papers reporting imaging findings on MRI for more than 3100 asymptomatic individuals and found that the prevalence approached those of people with symptoms in the back. For arthroscopic surgery for pain and degenerative changes in the knee, there is suspicion that they cause greater harms than benefit^32^, ^33^ and it is not possible to reliably identify subgroups of patients that benefit from the surgery.^34^ In the United States, spinal fusion surgery increased 600% between 1993 and 2011 mainly for the treatment of “degenerative disc disease”^35^ despite no evidence that fusion surgery is superior to well‐structured rehabilitation programs^36^ and despite no evidence showing an increase in prevalence of the condition.^37^ In addition, complex surgical procedures in the spine have greater risk of complications with no additional benefit over simpler surgical procedures,^38^ or indeed not performing the procedure at all.^35^

## REVERSING LOW‐VALUE CARE REQUIRES CHANGE AT ALL LEVELS

The shift in MSK guideline recommendations in recent decades to non‐pharmacological options as first‐line care and the identification of widespread low‐value care has brought challenges for healthcare systems everywhere. To bring care into line with best available guideline recommended evidence, behavior change at multiple levels is required including for patients, clinicians, organization of the clinical setting, and in the broader healthcare system. Before we can address the problems, implementation science researchers suggest that we need to understand the function of the current systems, including what is currently being done in clinical practice, how and why this is different from guideline recommendations, and what the potential solutions for bridging the gaps might be. Through systematic identification of barriers and facilitators to change, we can select intervention components that address each one specifically.^39^

Involvement of patients in planning and evaluation of health service delivery is recommended by the World Health Organization.^40^ This is motivated by a desire to design health systems that meet the needs of the end users. Olsson et al reviewed 34 studies from eight countries found evidence that patient involvement in designing service delivery is time consuming but can result in increased collaboration between healthcare providers and patients, increased motivation for organizational change, and changed clinical practice.^41^

At the clinician level, dissemination of information about best practice and clinical practice guidelines, and one‐off implementation efforts targeting individual clinicians does not generate sustained behavior change and high‐value care.^42^ In a Cochrane review including 30 original studies, Tzortziou Brown et al investigated the effectiveness of professional interventions for general practitioners that aim to improve the management of MSK conditions in primary care.^43^ They concluded that feedback on performance combined with guideline dissemination may lead to small improvements in guideline‐consistent GP behavior with regard to low back pain, while GP education on osteoarthritis pain and the use of influential physicians may lead to slight improvement in patient outcomes and guideline‐consistent behavior.^43^

At the health system level, authors of a recent systematic review identified 28 original studies evaluating the impact of implementing pathways designed to improve care as well as relieve overburdened secondary‐care centers. They found that service effiency could be improved through decreased wait times and appropriate use of consultant appointments, but that it was uncertain whether patient outcomes were improved.^44^ In addition, it is important to be aware that broader health systems issues such as reimbursement schemes and professional hierarchies influence what care is delivered and how.^45^, ^46^

Medical societies and health professional associations are addressing low‐value care, for example, via the establishment and support of the Choosing Wisely campaign. The aim of Choosing Wisely is to decrease healthcare waste and iatrogenesis, and one target of the campaign is imaging for low back pain.^47^ In Choosing Wisely professional bodies make public a core set of principles with recommendations to clinicians, patients, and other stakeholders regarding which interventions and services they should offer, and which not. Since its launch in 2012, Choosing Wisely has been established in more than 20 countries and has more than 100 participating professional associations^47^; however, large‐scale impact of this initiative has not yet been documented.^48^

## EFFECTIVE IMPLEMENTATION REQUIRES GOOD CONCEPTUAL AND THEORETICAL FRAMEWORKS

The use of theory to inform design of interventions can help to overcome what has been termed the “ISLAGIATT principle” (It Seemed Like A Good Idea At The Time), where inherent biases and personal beliefs, rather than theory, evidence, and a systematic approach, guide decisions.^49^, ^50^ Although research into how to best achieve behavior change has intensified in recent years, there is currently no strong basis for favoring one particular solution over another to overcome a particular low‐value care problem.^51^

Systematic assessment and selection of intervention components that address identified, modifiable barriers and enablers underpinned by an overarching framework is the most promising way forward.^52^ To date, however, implementation interventions to address low‐value care have been largely based on simple and mostly unstated models of human behavior, or in the case of the few theory‐based interventions, a systematic process has not been followed.^52^, ^53^, ^54^ Davis et al reviewed 235 evaluations of guideline dissemination and implementation studies and concluded that justification for the choice of implementation interventions was typically poor. Only 22.5% of interventions were based on a theory, and in just 6% the theory was explicit.^54^ Nonetheless, various conceptual and theoretical frameworks are applicable when aiming to replace low‐value care with better solutions.^52^ Importantly, there is a growing body of evidence demonstrating that interventions based on theory are more effective in changing behavior, even though few specific theories have been robustly tested in healthcare settings.^55^ Therefore the explicit use of theories and frameworks is encouraged based on available evidence, and guidance is available.^56^ Examples of commonly used theoretical implementation frameworks include Theoretical Domains Framework^57^, ^58^; the Consolidated Framework for Implementation Research (CFIR)^59^; the Exploration, Preparation, Implementation, Sustainment (EPIS) framework^60^; and the Re‐AIM framework.^61^

## EXAMPLES OF INTERVENTIONS DESIGNED TO FACILITATE HIGH‐VALUE CARE

There are attempts to implement programs that seek to address barriers to the uptake of guideline recommendations such as clinician knowledge and confidence and organizational barriers in the clinic. Examples include the PARTNER study where a model of service delivery that targets general practitioners and their patients seeking care for knee osteoarthritis are implemented.^62^ Patients are referred to a behavior change intervention aimed at promoting self‐management, and the GP is trained in delivering a tailored intervention focusing on exercise and weight loss.^62^ In the SOLAS project, physiotherapists are trained to deliver a group‐based intervention involving information and exercises supported by an e‐health leaning program for people with persistent back pain.^63^ The Danish GLA:D project is a structured program of patient education integrated with a group‐based supervised exercise for people with osteoarthritis of the knee or hip^64^, ^65^ or persistent and/or recurrent back pain. Outcomes from people receiving GLA:D are systematically followed in clinical registries and data are made public.^66^, ^67^ In the ALIGN cluster randomized trial, French et al compared the effectiveness of a tailored, multi‐faceted intervention based on guideline recommendations aiming at reducing inappropriate imaging referral and improve outcomes to passive dissemination of the guideline in people seeking care for acute back pain from physiotherapists and chiropractors in Victoria, Australia.^68^ They found that clinicians in the intervention group were more likely to provide advice about staying active, but there were no important differences in X‐ray referral and no difference in patient outcomes. Finally, Taylor et al compared the effectiveness of a participative group intervention that introduced cognitive behavioral approaches designed to promote self‐management to usual care and relaxation music. They found that it was not effective for reducing back‐related disability; however, some differences favoring the intervention group were found for depression and social integration, but not for pain intensity, self‐efficacy, or global perceived effect.^69^

## CONCLUSION

Low‐value care that wastes resources and harms patients is prevalent in health systems worldwide. Low‐value care affects the millions of people who suffer from MSK pain and disability who receive care that is contrary to recommendations in evidence‐based clinical practice guidelines. Low‐value care is provided by all health professions. If the trend of pervasive low‐value care is to be reversed, a concerted effort that involves all stakeholders including patients, clinicians, professional organizations, funders, decision makers, and health system administrators is needed. Such efforts should build on transparent investigation of barriers and enablers to best practice care delivery and designed using established conceptual and theoretical frameworks.

## CONFLICT OF INTEREST

Jan Hartvigsen declares that he is co‐developer of the GLA:D Back project, which is mentioned in this paper. He has no financial outcome from the project.

## Data Availability

No data available for this manuscript.
